# Shared decision making for prostate cancer screening: the results of a combined analysis of two practice-based randomized controlled trials

**DOI:** 10.1186/1472-6947-12-130

**Published:** 2012-11-13

**Authors:** Stacey L Sheridan, Carol Golin, Audrina Bunton, John B Lykes, Bob Schwartz, Lauren McCormack, David Driscoll, Shrikant I Bangdiwala, Russell P Harris

**Affiliations:** 1 Division of General Medicine and Clinical Epidemiology, School of Medicine, University of North Carolina, Chapel Hill, NC, 27599-7110, USA; 2 Sheps Center for Health Service Research, University of North Carolina, Chapel Hill, NC, USA; 3 Department of Health Behavior, Gillings School of Global Public Health, University of North Carolina, Chapel Hill, NC, USA; 4 RTI International, Research Triangle Park, NC, USA; 5 Department of Biostatistics, Gillings School of Global Public Health, University of North Carolina, Chapel Hill, NC, USA

## Abstract

**Background:**

Professional societies recommend shared decision making (SDM) for prostate cancer screening, however, most efforts have promoted informed rather than shared decision making. The objective of this study is to 1) examine the effects of a prostate cancer screening intervention to promote SDM and 2) determine whether framing prostate information in the context of other clearly beneficial men’s health services affects decisions.

**Methods:**

We conducted two separate randomized controlled trials of the same prostate cancer intervention (with or without additional information on more clearly beneficial men’s health services). For each trial, we enrolled a convenience sample of 2 internal medicine practices, and their interested physicians and male patients with no prior history of prostate cancer (for a total of 4 practices, 28 physicians, and 128 men across trials). Within each practice site, we randomized men to either 1) a video-based decision aid and researcher-led coaching session or 2) a highway safety video. Physicians at each site received a 1-hour educational session on prostate cancer and SDM. To assess intervention effects, we measured key components of SDM, intent to be screened, and actual screening. After finding that results did not vary by trial, we combined data across sites, adjusting for the random effects of both practice and physician.

**Results:**

Compared to an attention control, our prostate cancer screening intervention increased men’s perceptions that screening is a decision (absolute difference +41%; 95% CI 25 to 57%) and men’s knowledge about prostate cancer screening (absolute difference +34%; 95% CI 19% to 50%), but had no effect on men’s self-reported participation in shared decisions or their participation at their preferred level. Overall, the intervention decreased screening intent (absolute difference −34%; 95% CI −50% to −18%) and actual screening rates (absolute difference −22%; 95% CI −38 to −7%) with no difference in effect by frame.

**Conclusions:**

SDM interventions can increase men’s knowledge, alter their perceptions of prostate cancer screening, and reduce actual screening. However, they may not guarantee an increase in shared decisions.

**Trial registration:**

#NCT00630188

## Background

Prostate cancer screening is common despite uncertain evidence that screening is beneficial [1,2] and mounting evidence that screening may produce net harm [3]. According to national survey data [4], 75% of men age 50 and older in the US have been screened at some time during their lifetime. This is a striking fact, especially because only 63% of men in the United States have had colon cancer screening [4] and less than two-thirds of men have had screening and treatment for common cardiovascular risk factors [5-7], screening procedures which are known to save lives [8-11]. These high rates of prostate cancer screening in the face of current evidence have raised questions about how men understand and value prostate cancer screening compared to other common screening services for men. In the face of changing professional recommendations [3,12] they have also prompted a call for providers to use shared decision-making (SDM) [13] to assist men in deciding whether or not to undergo prostate cancer screening.

SDM is a process in which patients are involved as active partners in clinical decisions. It has been conceptualized in several different ways [14-16], but usually involves a process in which an individual learns about the seriousness of the illness; the benefits, harms, alternatives, and uncertainty of preventive or treatment options; weighs his or her values; and participates in the decision making process with the clinician in a shared role. The central feature of SDM is participation in the decision making process with the clinician (at least enough to abdicate a shared role if this is what they wish). This shared participation is what distinguishes SDM from informed decision making. It also is, theoretically, what allows doctors to clarify men’s understanding of key facts and relevant values, highlight the unique circumstances that might alter the decision for any individual, and add a considered perspective on the decision.

Although many have advocated SDM, most recent efforts to improve decision making about prostate cancer screening have focused on the development of decision aids and the promotion of informed decisions, with resultant improvements in knowledge and decision confidence and reductions in intent for screening and actual screening rates [17]. Few decision aids or other efforts [18-21] have provided the explicit skill building in patient-provider communication that might be expected to promote SDM for prostate cancer screening. Additionally, none that we are aware of have directed such skill building to both patients and providers to optimize the likelihood of a shared decision. In this manuscript, we explore the effects of an intervention to promote SDM for prostate cancer screening (including a video-based decision aid and researcher led coaching session for patients) that is supported by a 1-hour educational session for providers on outcomes including key components of SDM, intent for prostate cancer screening, and actual screening rates. We also secondarily explore the effects of framing prostate cancer screening in the context of other more clearly beneficial men’s health screening services.

## Methods

### Study overview

Between March 2005 and April 2006, we conducted two randomized controlled trials of the same prostate cancer screening intervention, alone or with additional information on two more clearly beneficial men’s health screening services (cardiovascular disease screening and colon cancer screening). We conducted each trial in a convenience sample from two practices (one academic and one community practice) within a single city (Chapel Hill, NC for the prostate only intervention and Greensboro, NC for the men’s health intervention). In both trials, we used the same educational video on highway safety (“Reducing Your Risk in a Crash” available at http://www.iihs.org/videos/default.html) as an attention control and employed identical implementation and measurement strategies to allow combining of data if no differences were noted in the patient outcomes of the two trials. In this manuscript we present the combined data from both trials. Our statistical considerations for combining the data are detailed in the methods below; results for the individual trials are presented separately in Additional file 1.

### Study sample and recruitment strategy

We invited all physicians from participating practices to attend a 1-hour educational session about prostate cancer screening and shared decision-making, provide informed consent, and join our study. Educational sessions were offered up to three times at each practice site with individual sessions offered to physicians who expressed interest, but were unable to attend these sessions.

Within each practice, we then recruited a convenience sample of men by identifying age eligible men from weekly schedules of physicians agreeing to participate. We contacted them via telephone to determine their eligibility. Men were eligible if they were aged 40–80 years old, had no prior history of prostate cancer, had been seen in the practice for at least one year, and if their physician had agreed to participate in the study. They were excluded if they were presenting for an acute medical visit or if they had evidence of a serious medical illness (e.g. intensive care hospitalization within the last 6 months, more than 2 hospitalizations in the last 6 months) because they and their physician would be unlikely to address preventive health issues. We invited eligible men to present for their regularly scheduled appointment an hour early to provide informed consent, enroll in the study, and complete study materials.

### Intervention structure and content

Our intervention consisted of 2 components designed by investigators (see Table 1): 1) a video-based decision aid for patients and 2) a coaching session for patients. The prostate specific content for our interventions was derived from a published systematic evidence review on prostate cancer screening [22]. In the men’s health version, this information was framed in the context of information about the prevalence of cardiovascular disease and colon cancer, the certain benefit of screening for these diseases, and the options and attributes of common screening tests and treatments for these diseases; see Part A of Additional file 2. Messages were pre-tested in three rounds of formative research (e.g. focus groups and cognitive and usability testing) in our target population [21] and then incorporated into our intervention.

**Table 1:** The intervention structure and content

**Intervention component**	**Purpose**	**Content**
Video for Patients	1) To describe key messages about prostate cancer screening	1) Key messages:
		· There are two kinds of prostate cancer—harmless and dangerous
		· A problem with the PSA test is that it leads some men with a harmless prostate cancer to get treatment that they do not need.
		· About half of all men who get treatment for prostate cancer will have permanent side effects
		· Men should decide whether the PSA test is right for them and talk with their doctor.
	2) To model the process of learning and deciding about prostate cancer screening	2) Modeling:
		· 4 men engage in an after-hours discussion with their physician
		· Each man participates in questioning and reasoning about screening
	3) To facilitate values clarification via a process of social matching with two men making opposite decisions using the same facts	Values Clarification:
		· Joe decides to get the PSA test after considering the facts
		· Frank decides NOT to get the PSA test after considering the facts
Coaching Session for Patients	1) To answer men’s questions about prostate cancer screening by providing a supplemental educational brochure	1) Key facts:
		· Location of the prostate
		· Characteristics of the PSA test
		· Characteristics of prostate cancer
		· Risk factors for prostate cancer
		· Treatment options and their side effects
	2) To help men clarify their values for screening by ranking and rating decisional attributes	2) Decisional Attributes:
		· Magnitude of the problem (e.g. prostate cancer)
		· Benefit in knowing one has prostate cancer
		· The (un)certainty of the PSA test
		· The (un)certainty of treatment outcomes
		· Worry about treatment side effects
	3) To help men prepare for a discussion with their doctor by delivering tailored messages about discussion barriers and by providing a pad on which to write their screening decision and any questions for their doctor	2) Barriers to Discussion:
		· Discomfort asking questions
		· Fear of expressing opinions
		· Difficulty interrupting the doctor
		· Difficulty disagreeing with the doctor
		· Worry about taking too much of the doctor’s time
		· Difficulty understanding medical jargon
		· Embarrassment asking questions
Education Session for Providers	1) To review the evidence for prostate cancer screening	1) Evidence:
		· Natural History of prostate cancer
		· Lack of clear benefit of prostate cancer screening
		· Certain harms of screening and early treatment
	2) To highlight the value of shared decision making for prostate cancer screening	2) Value of Shared Decision Making:
		· Ethical obligation to consider patient preferences in the face of uncertain outcomes

#### Video-based decision aid for patients

Our 12-minute video-based decision aid for patients was designed with three main objectives: 1) to provide the core information men would need to make an informed decision about prostate cancer screening, 2) to model the process of deciding whether or not to be screened, and 3) to help men begin to clarify their values and make a decision. The video showed four men engaged in an impromptu discussion about prostate cancer screening with their doctor. The discussion occurred after appointment hours in the clinic waiting room and allowed the doctor and men to exchange key information and reason together about the implications of each new piece of information provided. It ended with the doctor encouraging men to consider the facts and decide what they want. To help facilitate decision making and illustrate the individual nature of the decision, the doctor encouraged the men to consider the example of two men (Joe and Frank) who make opposite decisions using the same facts. To film the video, we employed an award winning video company: Insightment Video. Additionally, because messages were nuanced, we employed actors to deliver the script. Core video content is shown in Table 1.

#### Counselor delivered coaching tool for patients

Our 8-minute coaching tool was modeled after an effective coaching tool by Kennedy and colleagues [23] and employed scripted materials delivered by a trained health counselor. It had three main objectives: 1) to answer men’s additional questions about prostate cancer screening, 2) to help men further clarify their values for prostate cancer screening, and 3) to prepare men to discuss prostate cancer screening with their doctor (see Additional file 2).

We addressed additional questions we anticipated men might have through a supplemental brochure. The brochure reinforced and expanded on content presented in the video and included the following topics: the location of the prostate, the characteristics of prostate cancer and the PSA test, the risk factors for prostate cancer (age, family history, race), and treatment options for prostate cancer (radiation and surgery), including their side effects. Like other materials, brochures were framed to discuss prostate screening alone or broader issues of men’s health. Copies of relevant brochures were given to each man to take home.

We helped men clarify their values for prostate cancer screening using a process in which men rated and then ranked the relative importance of several factors in their decision making. The decision factors included: 1) the chances of dying from prostate cancer, 2) the need to know whether or not one has cancer, 3) the certainty of the diagnosis provided by the PSA test, 4) the certainty of benefit from screening and treatment, and 5) worry over treatment side effects. We first asked men to read a series of two opposing statements about each decision factor and choose which statement best represented their own feeling about that factor. For instance, for the factor “need to know”, men chose between the following two statements: 1) I *don’t like the idea* of having prostate cancer and not knowing it even though more prostate cancers are harmless than dangerous, or 2) I’m *ok* with not knowing I have prostate cancer because more prostate cancers are harmless than dangerous. We then asked men to rank which three of their five chosen statements most affected their decision making and state whether or not they intended to be screened or were still unsure and needed to consider it further. To facilitate the process of rating and ranking, all statements were written on laminated cards that could be rearranged for consideration.

To help men prepare for discussions about prostate cancer screening with their doctor, we first asked men to consider how involved they’d like to be in decision making about prostate cancer screening. We then delivered scripted counseling on how to address barriers to communication. Men endorsed barriers from the following list: 1) discomfort asking the doctor questions; 2) fear of upsetting the doctor by expressing opinions; 3) difficulty interrupting the doctor; 4) concern that it is not one’s place to disagree with the doctor; 5) worries of taking up too much of the doctor’s time; 6) difficulty understanding doctors who use medical jargon; and 7) embarrassment over not knowing things and having to ask the doctor questions. They then received counseling on as many barriers as they endorsed. Following counseling, men received a “list pad” which summarized key messages and encourage men to write down questions to ask their doctor.

### Intervention and survey delivery and the procedure of randomization

The patient intervention and accompanying surveys were delivered to participants prior to regularly scheduled medical appointments in a private room in each practice. After providing informed consent and completing a baseline survey, eligible men were randomized within practices to receive one of the SDM interventions or a highway safety control video. Randomization used computer-generated random numbers that were sealed in opaque envelopes. Men in the intervention groups watched one of our informational videos and then were guided through a coaching session by a trained research assistant. At the end of the coaching session, men were given a summary sheet of their opinions on prostate cancer screening to share with their physician. Men in the highway safety group viewed the highway safety video, but received no accompanying coaching session. After completion of these interventions, all men completed a 2^nd^ questionnaire to assess immediate changes in their knowledge and attitudes about prostate cancer screening. Men then proceeded to their visit with their physician (who was notified only about patients’ participation, but not group assignment) and, on completion of their visit, filled out a final questionnaire about the content of their visit with their physician.

Physicians in our study were also asked to complete a baseline questionnaire so that we could capture their demographics, knowledge, and attitudes about screening.

### Measurement

We measured the main effect of our SDM interventions by examining three key components of shared decision making. We then, secondarily, measured men’s decision for screening following their doctor’s visit, and their actual screening rates at 9 month follow-up.

#### Measuring the key components of shared decision-making

To assess the effects of our intervention, we measured the following three outcomes: 1) perception that prostate screening requires a personal decision, 2) knowledge about prostate cancer and prostate cancer screening, and 3) participation in the decision-making, including both shared participation and participation at their preferred level [16].

##### Perception that prostate cancer screening requires a decision

To measure perception that prostate cancer screening requires a decision, we assessed men’s agreement with the following statement: “it is okay to decide not to have a PSA test after learning the facts.” This question is one of three questions in the “PSA is a Decision” score (alpha 0.76), is highly correlated with overall scale results [24], and was been shown to be sensitive to intervention effects in community study of PSA decision making that was conducted by our research team. Responses were scored on a 5-point Likert scale (strongly agree to strongly disagree) and categories were collapsed to create a dichotomous variable showing men’s agreement (strongly agree or agree) with this statement.

##### Knowledge about prostate cancer screening

To measure knowledge about prostate cancer screening, we designated four key knowledge questions, which captured the core messages in our intervention and represented knowledge we felt was essential to making a good decision about prostate cancer screening. These questions highlighted the benign natural history of most prostate cancers and the high likelihood of side effects with treatments delivered for prostate cancer detected by PSA screening: 1) “Some men can live long lives with prostate cancer,” 2) “most men diagnosed with prostate cancer die of something else,” 3) “problems with sexual function is a common side effect of prostate cancer treatments,” and 4) “problems with urination is a common side effect of prostate cancer treatments.” These true-false questions were adapted from questions used in other trials of prostate screening [25]. If men agreed with all of these statements, they were considered to have the key knowledge necessary for an informed decision.

##### Participation in decision-making

To measure men’s preferred participation in decision-making, we adapted a widely employed measure for use in a survey format [26]. Our 5-point Likert question asked men “how much would you like to be involved in the decision about whether or not to get the PSA test,” with responses on a continuum between making the final decision themselves or having the doctor make the decision himself. For analysis, we collapsed answers into three categories (patient decision (I decide); shared decision (I decide after considering the doctor’s opinion + doctor and I decide together + doctor decides after considering my opinion); and doctor’s decision (doctor decides). We also specifically assessed who reported shared rather than independent (either doctor or patient) decision making.

To measure men’s actual participation, we asked a similar question: “how much were you involved in the decision about whether or not to get a PSA test today?” Responses were provided on the same Likert scale with one additional response category: we talked about the PSA test, but didn’t make a final decision. We collapsed this response with shared decisions, consistent with several shared decision making models calling for patients and physicians to make or delay decisions.

We then examined the proportion of shared decisions and compared men’s preferred participation after the intervention with their stated participation during their visit to determine whether men participated in decision-making at their desired level.

#### Measuring intent for screening

We measured men’s intent for screening before and after the intervention using a single item question: “In the next 12 months, do you plan to get a PSA test?”

#### Measuring actual screening rates

We determined actual prostate cancer screening rates in two ways. First, we asked men immediately following their visit with their clinician “Did you get a PSA today?” Second, we reviewed men’s medical records approximately nine months following their study visit to determine whether they’d followed through with their original decision about prostate cancer screening.

#### Measuring baseline characteristics that might affect prostate cancer decisions and screening

Because of our small sample size and the possibility that randomization may have resulted in an unequal distribution of patient characteristics across groups, we measured several characteristics of men and physicians that might affect patients’ decision making and actual screening rates. Men’s characteristics included self-reported age, race, education, marital status, usual source of care, family history of prostate cancer, prior history of PSA tests, abnormal PSA tests, and prostate biopsies. We also measured men’s certainty with their plans for prostate cancer screening using the 3-item uncertainty subscale from O’Connor’s Decisional Conflict Scale [27]. Physician’s characteristics included self-reported age, race, gender, prior history of PSA testing, preferred approach to prostate cancer screening (e.g. do it, discuss it, or don’t discuss/don’t do), preference for SDM, and perceptions that their patients are involved enough to influence decisions.

### Statistical analysis

To assess the randomization process, we compared the baseline characteristics of subjects assigned to the SDM interventions with those assigned to the control group. Within each of our two randomized trials, we then examined the effect of our interventions on the key components of SDM and on men’s decisions and actual screening rates. Finding no difference in the patient outcomes between the two individual trials (see Additional file 1), we then combined data from the two trials for all subsequent analyses, accounting for the random effects of practices as is done in meta-analysis.

In combined analysis, we examined the effect of the SDM interventions on the key components of SDM by comparing *differences* in the key components of SDM post-intervention between study arms. To deepen our understanding of intervention effects, we also examined *changes* in the key components of SDM across study arms (see Additional file 3). We used Pearson’s chi-square tests (SAS Statistical Software, Cary, NC) to evaluate whether the interventions significantly affected men’s decision for screening, and their actual screening rates. For these unadjusted analyses, we report absolute differences and confidence intervals between the intervention and control groups to allow interpretation of the magnitude of clinical effect.

We then used mixed effects logistic regression models to examine whether the intervention affected outcomes after adjusting for the random effects of both practices and physicians and for the baseline differences among intervention groups. For each model, we adjusted only for conceptually relevant covariates that were 1) related to the outcome in bivariate analysis, and 2) changed the outcome of the model by more than 10% when added in forward stepwise regression. Because results varied with adjustment, we report both unadjusted and adjusted analyses. We calculated relative risks from odds ratios using standard formulas [28]. When adjusted analyses involved dichotomous covariates, we present adjusted relative risks based on clinically relevant covariate responses (e.g. plans for PSA = probably or definitely yes; history of PSA =yes)

### Sample size calculation

Sample size was primarily driven by budgetary and practical considerations. We expected the 128 individuals would provide adequate power for the overall comparison of interventions arms for most outcomes of interest.

## Results

We recruited 36 physicians to participate in our study (11% of eligible physicians at the academic practice in Chapel Hill; 100% of eligible physicians at the community practice in Chapel Hill; 100% at the academic practice in Greensboro; 75% at the community practice in Greensboro). 28 physicians saw patients enrolled in our study and are therefore included in our analysis; their characteristics are shown in Table 2. In general, these physicians were young, predominantly male, and preferred a shared approach to decision-making about prostate cancers screening. Most of the included male physicians had never been screened for prostate cancer.

**Table 2:** Physician characteristics (n = 28)

**Mean age (range)**	**36 (27 to 57)**
Male Gender	54%
Race	
White	64%
African-American	18%
Other	18%
History of PSA Screening *	
Ever	33%
Never	67%
Approach to Prostate Cancer Screening	
Do it	4%
Discuss it	71%
Don’t discuss/don’t do it	25%
Prefer Shared DM for PCS	79%
Patients Involved Enough to Affect Decision	
Almost always	11%
Very often	21%
Often	36%
Seldom	25%
Almost never	7%

*Males Only.

**Figure 1:**
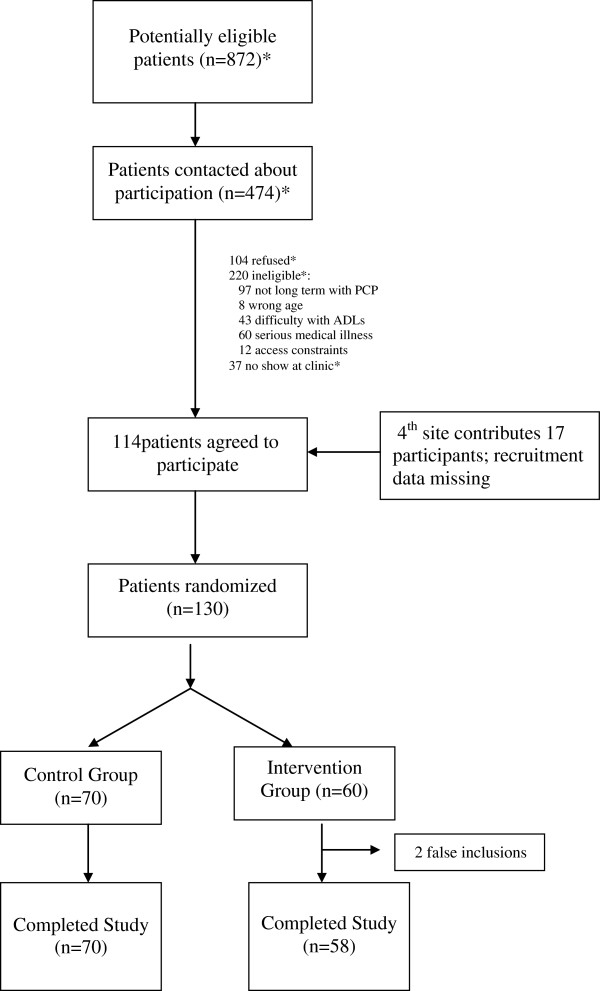
Recruitment and Enrollment.

Our patient recruitment is shown in Figure 1. In total, we recruited and enrolled 130 patients (70 in the control group and 58 in the intervention group) and 128 completed the study. The baseline characteristics of included patients are shown in Table 3. Those in the control and intervention groups were similar with regard to age, race, educational status, marital status, and usual source of care. Participants in the control group, however, reported more prior screening, more discussions about prostate cancer screening in the last 12 months, and more plans for screening in the next 12 months. Participants in the control group were additionally slightly less likely to consider prostate cancer screening a decision and slightly more likely to have key knowledge about prostate cancer screening.

**Table 3:** Baseline patient characteristics

	**Control**	**Intervention**
	**(n = 70)**	**(n = 58)**
Mean age (range)	58 (41 – 74)	57 (41 – 78)
White race	56%	53%
Education:	70%	65%
At least some college		
Marital Status:	59%	64%
Married		
Personal Doctor	96%	96%
FH of Prostate Cancer	13%	4%
Discussed PSA with MD in last 12 months	51%	41%
Prior MD recommendation for screening	22%	14%
Previous PSA Screening (ever)	59%	44%
Previous Abnormal PSA	10%	7%
Plan for PSA Screening in next 12 months	80%	69%
Think PSA Screening is a Decision	17%	34%
Have Key Knowledge about PSA Decision*	10%	3%
Preferred Participation in DM:		
I decide	16%	25%
Share decision	77%	71%
MD decides	7%	4%
Decisional Conflict, uncertainty score (sd)†	1.9 (0.8)	1.9 (0.8)

*Key knowledge: Agreement with the following four statements: 1) Some men can live long lives with prostate cancer, 2) most men diagnosed as prostate cancer die of something else, 3) problems with sexual function is a common side effect of prostate cancer treatments, and 4) problems with urination is a common side effect of prostate cancer treatments.

**†Decisional Conflict, uncertainty score:** Mean agreement on 1–5 scale for the following 3 questions: 1) The decision is easy for me to make, 2) I am sure what to do in this decision, 3) It is clear what choice is best for me.

### The effect of the intervention on key components of decision making

In the post intervention period, more men in the intervention group than in the control group agreed that prostate cancer screening is a decision (see Table 4; absolute difference: 41%, 95% CI 25% to 57%; fully adjusted RR 2.79, 95% CI 1.96 to 3.47) and had the key knowledge necessary to make a good decision (absolute difference: 34%, 95% CI 19% to 50%; fully adjusted RR 4.28, 95% CI 2.30 to 6.45). Men in the intervention group, however, were no more likely to participate in shared decision making (absolute difference post-intervention: -3%, 95% CI −21% to +15%; fully adjusted RR 0.96, 95% CI 0.67 to 1.15) or decision making at their preferred level (absolute difference post-intervention: -5%, 95% CI −24% to +13%; fully adjusted RR 0.92, 95% CI 0.64 to 1.11) than men in the control group.

**Table 4:** The effect of the intervention on key components of decision making

	**Control, % (n)**	**Intervention, % (n)**	**Unadjusted absolute difference**	**Unadjusted RR**	**Adjusted RR**	**Adjusted RR**	**Adjusted RR**
	**(n = 70)**	**(n = 58)**	(95% CI)*	**(95% CI)**	**(95% CI)†**	**(95% CI) ‡**	**(95% CI) §**
Overall							
**% of Men Agreeing PSA is a Decision, post-intervention:**	23% (16)	64% (37)	41% (25 to 57%)	2.79 (1.74 to 4.47)	3.57 (2.33 to 7.69)	2.79 (1.96 to 3.47)	---∥
**% Men Having Key Knowledge, post intervention:**	13% (9)	47% (27)	34% (19 to 50%)	3.63 (1.86 to 7.08)	4.55 (2.38 to 33.3)	4.28 (2.30 to 6.45)	---∥
	**Among Men Who Talked with Their Doctor about PSA Testing**						
	**Control, % (n)**	**Intervention, % (n)**	**Absolute Difference**		**Adjusted RR**	**Adjusted RR**	**Adjusted RR**
	**(n = 51)**	**(n = 38)**	(95% CI)*		**(95% CI)†**	**(95% CI)‡**	**(95% CI) §**
**% of Men Reporting Shared Decisions, post-visit:**	76% (39/51)	74% (28/38)	−3% (−21% to +15%)	0.96 (0.76 to 1.23)	1.01 (0.76 to 1.27)	0.96 (0.67 to 1.15)	---∥
**% of Men Reporting Participation at preferred level, post-visit**	76% (39/51)	71% (27/38)	−5% (−24% to +13%)	0.92 (0.72 to 1.20)	0.93 (0.74 to 1.23)	0.92 (0.64 to 1.11)	---∥

*Pearson Chi-square tests.

† Adjusted for random effects of physician.

‡ Adjusted for random effects of physician and practice.

§Adjusted for random effects of physician and practice + family history of prostate cancer, history of PSA testing, receipt of physician recommendation for testing, current plans for PSA testing, and patient approach to discussing PSA testing at next visit (as applicable after stepwise regression).

∥Not reported because no baseline variables retained in model during modeling process.

### The effect of the intervention on intent for screening and actual screening rates

Immediately after receiving our intervention, 79% of men in the control group reported plans for PSA screening in the next 12 months compared with 45% of men in intervention group (see Table 5; absolute difference: -34%, 95% CI −50% to −18%; fully adjusted RR 0.18, 95% CI 0.06 to 0.48). Concordantly, immediately following their doctor’s visit, fewer men in the intervention group than the control group reported they had been screened (unadjusted absolute difference −21%, 95% CI −38% to 4%; fully adjusted RR 0.42, 95% CI 0.14 to 1.24). Screening rates by chart review at 9 months showed similar results (unadjusted absolute difference −22%; 95% CI −38 to −7%; fully adjusted RR 0.79, 95% CI 0.50 to 0.97) with rates remaining appreciably lower in both groups than men’s stated plans for screening. 

**Table 5:** Effect of the intervention on men’s decisions and actual screening rates

	**Control, % (n)**	**Intervention, % (n)**	Unadjusted Absolute Difference *	**Unadjusted RR**	**Adjusted RR**	**Adjusted RR**	**Adjusted RR**
	**(n = 70)**	**(n = 58)**	**(95% CI)**		**(95% CI)** †	**(95% CI)** ‡	**(95% CI)** §
**Intent for Screening Post Intervention**	79% (55)	45% (26)	−34% (−50% to −18%)	0.57 (0.42 to 0.78)	0.46 (0.34 to 0.73)	0.57 (0.35 to 0.81)	0.18 ( 0.06 to 0.48)∥
**Patient reported screening after clinical visit**	31% (16)	11% (4)	−21% (−38% to −4%)	0.44 (0.17 to 1.08)	0.27 (0.12 to ∞)	0.43 (0.16 to 0.96)	0.42 (0.14 to 1.24)∥
**Actual Screening at 9 months**	41% (29)	19% (11)	−22% (−38 to −7%)	0.45 (0.25 to 0.83)	0.43 (0.26 to 1.41)	0.42 (0.20 to 0.81)	0.76 (0.50 to 0.97)**

* Pearson’s chi-square tests.

†Adjusted for random effects of physician.

‡Adjusted for random effects of physician and practice.

§Adjusted for random effects of physician and practice and for family history of prostate cancer, history of PSA testing, receipt of physician recommendation for testing, current plans for PSA testing, and patient approach to discussing PSA testing at next visit (as applicable after stepwise regression).

∥Adjusted only for plans for PSA testing; other covariates dropped out of model.

** Adjusted only for History of PSA test; other other covariates dropped out of model.

## Discussion

In a combined analysis of two randomized controlled trials promoting SDM for prostate cancer screening, the intervention had mixed effects on the key components of SDM: it increased men’s perception that screening is a decision and men’s knowledge about prostate cancer screening, but had no effect on men’s preferred or actual participation in shared decisions. Men who were exposed to the intervention were significantly less likely to plan to get screened in the next 12 months and actually get screened according to chart review at 9 months.

Our findings suggest the ability of SDM interventions to increase men’s knowledge and alter their intent for prostate cancer screening, but additionally highlight the complexities of promoting SDM: namely helping men participate equally with their clinician, or, at least, participate at a level at which they desire. We designed our intervention to prepare both men and doctors for SDM. We encouraged men to consider the facts, decide what they wanted, and talk with their doctor and, then, provided advice on overcoming barriers to talking with their doctor. Additionally, we provided doctors with one session of education on the rationale and recommendations for SDM. With these efforts, we increased the proportion of men who perceived prostate cancer screening to be a decision and had adequate knowledge and decreased intent for screening. We did not, however, increase the proportion of men who shared a decision with their doctor or participated at the level they desired. There are four possible explanations for these findings: men either a) talked with their doctor and agreed that a primarily independent decision was appropriate, b) didn’t see the value in equal participation when they had already received the relevant facts, clarified their values, and considered what they wanted, c) didn’t (despite our intervention) know how to engage the doctor for equal participation when they wanted to, or d) had a doctor who (despite our educational session) didn’t engage in shared decisions.

An important question given our results is whether there is an added benefit of sharing the decision with a doctor beyond getting the facts and considering one’s own values. The theoretical benefit of sharing the decision is that it allows doctors to clarify men’s understanding of key facts and relevant values, highlight the unique circumstances that might alter the decision for any individual, and add a considered perspective on the decision. Several of these functions may not be necessary when decision aids are available, particularly if men are known to be health literate and the decision aid allows for deliberation on values and preferences. Instead, independent decision making (with the doctor in a supporting role) may sometimes be appropriate and improve men’s self-efficacy for following through with a decision [29]. Whether a shared decision adds benefit over an informed decision is an empirical question that should be addressed in future research. An observational analysis of the outcomes that result when decisions are either shared, made at a level which men desire and feel comfortable, or made without regard to men’s preferences, would be helpful in resolving questions about SDM (especially if the analysis is focused on outcomes such as value concordance and adherence to decisions). Additionally, an analysis of the frequency of decisions in which clinicians’ input might substantially alter the decision making process would be helpful.

Future research should also attempt to measure the relative contribution of each component included in SDM interventions. We are unclear which components of our intervention had the most impact on decision making and screening outcomes or of the independent value of our novel coaching tool. Testing the relative effect of various components will aid construction of new interventions and refinement of existing ones. Future work might also explore other intervention components or component content that might make SDM interventions more effective in promoting shared decisions. For instance, researchers might explore alternate messages about how to share a decision (i.e. men should clearly state their preference for decision making; or men should ask their doctor whether their unique circumstances should alter the decision making process). Researchers might also explore stronger interventions for physicians (i.e. to help them identify patients’ preferences and encourage question asking). A few simple changes might refocus men and their physicians on the value of sharing decisions and facilitate the process, thereby promoting such decisions.

In considering future directions, researchers should also consider how to improve on the methods we used. First, our study sample included a convenience sample of men, who may have exhibited differences from the source population from which they were sampled. Future work should consider random sampling from the source population. Second, despite randomization, the small size of our study resulted in differential distribution of confounders among study groups. We controlled for this in multivariate analysis, but recognize the potential that unmeasured confounders may have affected our results. Future work should employ larger sample sizes to ensure the success of randomization. Third, because we randomized at the patient level, physicians saw patients in both the intervention and control groups, creating the possibility for contamination. Future work with greater resources should consider randomization of physicians or practices. Fourth, other factors may have biased our effect size. Providers may have altered their behavior merely because they knew they were being watched. Similarly, patients or providers may have altered their survey responses because based on their study assignment (or assessment thereof). Fifth, our measures of the key components of SDM (including our measures of knowledge and patient participation) haven’t been formally validated. Future work should consider a full assessment of the validity and reliability of our measures or alternate methods of measuring knowledge [25] and patient participation [30] to ensure the validity of conclusions. More extensive knowledge and participation measures and/or newer measures of informed and shared decision making might draw different conclusions [31-33]. Finally, the generalizability of our sample may be limited. We enrolled a convenience sample of men from 4 conveniently located academic and community practices in two cities in North Carolina. Approximately 45% of men had engaged in discussions with their physician in the last 12 months, suggesting they may be a more educated and activated group than most. Sampling from more diverse practices in diverse locations would improve the generalizability of data. Further, we addressed only 1 clinical decision (e.g. prostate cancer screening). To the extent that preferred and actual involvement in decision making differs across clinical decisions, the benefits of shared decision making interventions similar to ours (i.e. including a decision aid, coaching session, and physician education session) may differ across decisions and should be tested.

## Conclusions

Despite limitations, we believe our study makes important contributions to—and raises critical questions about--our understanding of SDM. SDM interventions may not guarantee increases in shared decisions, but nonetheless change clinical outcomes. More work is needed to determine the added value of a shared decision (above and beyond an informed decision), and, if of added value, how to best promote a shared decision. Parts of this work were previously presented at the National Society of General Internal Medicine meeting in May 2006.

## Competing interests

The authors declare that they have no competing interests.

## Authors’ contributions

SS obtained funding, participated in the conception of the study, performed data analysis, and led manuscript drafting and revising. CG and RH obtained funding, participated in the conception of the study, and performed data analysis. AB and JBL led the data collection process. BS performed data analysis. LM and DD obtained funding and participated in the conception of the study. KB performed data analysis and provided statistical expertise. All authors reviewed the manuscript for critical revisions and have approved the final manuscript.

## Pre-publication history

The pre-publication history for this paper can be accessed here:

http://www.biomedcentral.com/1472-6947/12/130/prepub

## Supplementary Material

Additional file 1 Results by City. Click here for file

Additional file 2 Coaching Tool Detail. Click here for file

Additional file 3 The Effect of the Intervention on Changes in Key Components of Decision Making. Click here for file
