# Supplementary material for: Shared decision making for prostate cancer screening: the results of a combined analysis of two practice-based randomized controlled trials
Source: BMC Med Inform Decis Mak. 2012 Nov 13;12:130. doi: 10.1186/1472-6947-12-130 (PMC3582602; doi:10.1186/1472-6947-12-130)
Supplement: Additional file 1 — Results by City. [file 1472-6947-12-130-S1.docx]

**Additional file 1**

**Figure 1 - Recruitment and Enrollment by City**

Patients contacted about participation (n=212)*

30 refused*

133 ineligible*:

84 not long term with PCP

4 wrong age

14 difficulty with ADLs

25 serious medical illness

6 access constraints

13 no show at clinic*

Patients randomized

(n=53; 36 from MC)

Intervention Group (n=26)

Control Group (n=27)

Completed Study (n=27)

Completed Study

(n=26)

Potentially eligible patients in Greensboro (Men’s Health; n=313)*

Patients contacted about participation (n=262)

74 refused

87 ineligible:

13 not long term with PCP

4 wrong age

29 difficulty with ADLs

35 serious medical illness

6 access constraints

24 no show at clinic

Patients randomized

(n=77)

Intervention Group (n=34)

Control Group (n=43)

Completed Study (n=43)

Completed Study

(n=32)

Potentially eligible patients in Chapel Hill (Prostate Only; n=559)

*Data missing for non-academic site

2 false inclusions

**Table 1. Baseline Characteristics, By City**

|  | Prostate Only (n=75) | | Men’s Health (n=53) | |
| --- | --- | --- | --- | --- |
|  | Control  (n=43) | Intervention  (n=32) | Control  (n=27) | Intervention  (n=26) |
| Mean age (range) | 58 (41-74) | 58 (41-78) | 59 (41-72) | 55 (41-72) |
| White race | 65% | 72% | 41% | 31% |
| Education:  At least some college | 86% | 91% | 44% | 32% |
| Marital Status:  Married | 74% | 84% | 37% | 38% |
| Personal Doctor | 93% | 97% | 100% | 96% |
| FH of Prostate Cancer | 10% | 6% | 19% | 0% |
| Discussed PSA with MD in last 12 months | 60% | 50% | 37% | 31% |
| Prior MD recommendation for screening | 26% | 16% | 15% | 12% |
| Previous PSA Screening (ever) | 69% | 52% | 44% | 35% |
| Previous Abnormal PSA | 12% | 10% | 7% | 4% |
| Plan for PSA Screening in next 12 months | 74% | 66% | 89% | 73% |
| Preferred Participation in DM:  I decide  I decide, MD input  Share decision  MD decide, my input  MD decides | 19%  33%  40%  5%  5% | 26%  39%  35%  0%  0% | 11%  37%  37%  0%  11% | 23%  35%  31%  4%  8% |
| Previous Exposure to Community Materials | NA | NA | 23% | 7% |

**Table 2 - The Key Components of a Shared Decision**

|  | **Prostate Only** (n=75) | | | **Men’s Only** (n=53) | | |
| --- | --- | --- | --- | --- | --- | --- |
|  | **Control**  (n=43) | **Intervention**  (n=32) | **Absolute Difference**  **(95% CI)** | **Control**  (n=27) | **Intervention**  (n=26) | **Absolute Difference**  **(95% CI)** |
| **% of Men Agreeing PSA is a Decision, post-intervention:** | 26% | 69% | 43%  (22% to 63%) | 19% | 58% | 39%  (15% to 63%) |
| **% Men Having Key Knowledge, post intervention:** | 17% | 65% | 48%  (27% to 68%) | 7% | 27% | 19%  (0% to 39%) |
| **% of Men Reporting Shared Decisions, post-visit:** | 73% | 65% | -7%  (-33% to 18%) | 83% | 83% | 0%  (-24% to 24%) |
| **% of Men Reporting Participation at preferred level, post-visit** | 73% | 65% | -7%  (-33% to -18%) | 83% | 78% | -5%  (-31% to 20%) |

**Table 3 - Men’s Plans and their Actual Screening Rates**

|  | **Prostate Only (n=75)** | | | **Men’s Health (n=53)** | | |
| --- | --- | --- | --- | --- | --- | --- |
|  | **Control**  **(n=43)** | **Intervention**  **(n=32)** | **Absolute Difference (95% CI)** | **Control**  **(n=27)** | **Intervention**  **(n=26)** | **Absolute Difference (95% CI)** |
| **Intent for Screening Post Intervention** | 74% | 41% | -34%  (-55% to -12%) | 85% | 50% | -35%  (-58% to -12%) |
| **Patient reported screening after clinical visit** | 32% | 15% | -17%  (-40% to 5%) | 26% | 11% | -15%  (-40% to 9%) |
| **Actual Screening at 9 months** | 44% | 19% | -25%  (-45% to -5%) | 67% | 33% | -17%  (-42% to -6%) |
