# Supplementary material for: Shared decision making for prostate cancer screening: the results of a combined analysis of two practice-based randomized controlled trials
Source: BMC Med Inform Decis Mak. 2012 Nov 13;12:130. doi: 10.1186/1472-6947-12-130 (PMC3582602; doi:10.1186/1472-6947-12-130)
Supplement: Additional file 2 — Coaching Tool Detail. [file 1472-6947-12-130-S2.pdf]

Additional file 2. Coaching Tool Detail

A. Answering Men's Questions

One purpose of our coaching tool was to answer men's questions about prostate cancer screening or, if appropriate, broader men's health. We did this using supplemental brochures (see below and on following pages) which reiterated and expanded on the content of our video-based decision aid.

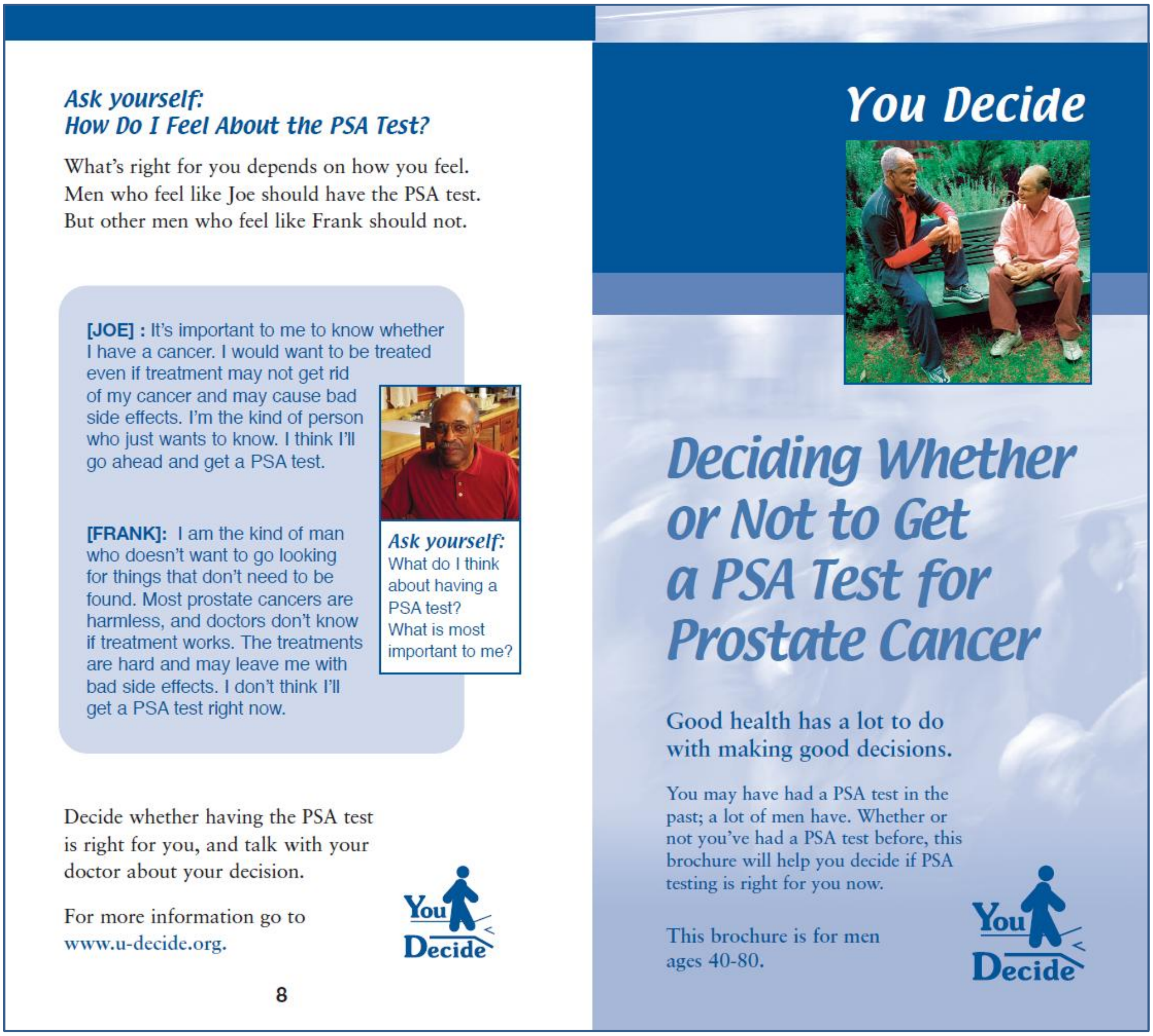

Ask yourself: How Do I Feel About the PSA Test?

What's right for you depends on how you feel. Men who feel like Joe should have the PSA test. But other men who feel like Frank should not.

[JOE] : It's important to me to know whether I have a cancer. I would want to be treated even if treatment may not get rid of my cancer and may cause bad side effects. I'm the kind of person who just wants to know. I think I'll go ahead and get a PSA test.

[FRANK]: I am the kind of man who doesn't want to go looking for things that don't need to be found. Most prostate cancers are harmless, and doctors don't know if treatment works. The treatments are hard and may leave me with bad side effects. I don't think I'll get a PSA test right now.

Ask yourself:
What do I think about having a PSA test?
What is most important to me?

Decide whether having the PSA test is right for you, and talk with your doctor about your decision.

For more information go to www.u-decide.org.

You Decide

8

You Decide

Deciding Whether or Not to Get a PSA Test for Prostate Cancer

Good health has a lot to do with making good decisions.

You may have had a PSA test in the past; a lot of men have. Whether or not you've had a PSA test before, this brochure will help you decide if PSA testing is right for you now.

This brochure is for men ages 40-80.

You Decide

Prostate Specific Antigen (PSA) and the PSA Test

The Prostate Specific Antigen (or PSA) test is a blood test that can help to find prostate cancer. PSA is a natural substance made by the prostate, a small gland that only men have. The prostate is located between the bladder (that holds your urine) and your penis. The urine tube (urethra) runs through the prostate.

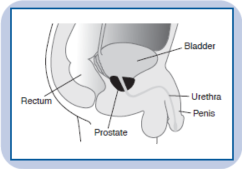

The PSA test has some problems. A small amount of PSA in the blood is normal. Higher amounts of PSA can come from prostate cancer or from having an enlarged prostate with no cancer (a condition that many men have after age 50). That is why most men with a high PSA level don't have cancer – they have an enlarged prostate instead. Also, some prostate cancers don't make much PSA – so some men have prostate cancer even though they have a normal PSA test.

There Are Two Types of Prostate Cancer – Harmless and Dangerous

The prostate changes as men get older. In some men, the prostate gets larger. Another change is that some normal parts of the prostate start to look like cancer. Even though they look like cancer, these parts don't act like cancer – they never cause any problems. These are called "harmless" prostate cancers. Men can live long, normal lives with a harmless prostate cancer. Research shows that harmless prostate cancers grow so slowly that they will never become dangerous.

Most prostate cancers are harmless. If you think of 10 men who have prostate cancer, about 6 have harmless cancer. Only about 4 of these ten men have the dangerous type of prostate cancer.

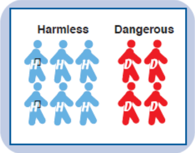

How can we find prostate cancer early? What are its symptoms?

Early prostate cancer doesn't have any symptoms – so that's not a good way to find it. The best way would be if doctors could just find dangerous prostate cancers and leave the harmless ones alone. But the PSA test finds both kinds of prostate cancers – harmless and dangerous – and doctors can't tell which one a man has.

Different Treatments for Prostate Cancer

If your PSA test level is high, your doctor will probably send you to a specialist. The specialist will do a "biopsy" of your prostate (a biopsy is when they stick small needles in your prostate to take samples to do more tests.)

If the biopsy shows cancer, it means that you would probably get treatment. Common treatments are:

- surgery (radical prostatectomy) – a major operation that removes the entire prostate, and
- external radiation therapy – burning a part of the prostate by using radiation.

Treatment may or may not help men with dangerous prostate cancer live longer. Doctors are still studying this.

Some other treatments are used less often, like internal radiation therapy (brachytherapy), where a doctor performs surgery to place small radioactive pellets inside or near the cancer to destroy cancer cells. You may have also heard of "watchful waiting." This is not a common treatment option except in men over age 70, and it involves monitoring the patient's prostate cancer with regular testing and treating it only if the prostate cancer causes symptoms or shows signs of growing.

Treatment for Prostate Cancer Can Cause Permanent Side Effects

Doctors don't know which treatment for early prostate cancer is best or if any of today's treatments help men live longer.

About half of men who get treatment for prostate cancer will have permanent side effects, including problems having an erection (also called "impotence") and problems holding their urine (also called "incontinence").

Doctors do know that about half of all men who get surgery or radiation treatment will have permanent side effects from that treatment.

A problem with the PSA test is that it leads some men with harmless prostate cancer to get treatment that they don't need.

Because doctors can't tell whether a prostate cancer is the dangerous or the harmless kind, they treat almost everyone. This means that some men who only had a harmless prostate cancer will end up with bad side effects.

Risk Factors for Prostate Cancer

Although all men are at risk for prostate cancer, some men are at greater risk.

> The men who need to decide about having a PSA are between the ages of 50 and 70.

Age. The chance of getting prostate cancer for men under 50 is very low—these men don't have to worry about getting a PSA test. Men's chances of dying from prostate cancer go down after age 70. After age 70, he has a much higher chance of dying from something else. Doctors agree that men over age 70 don't need a PSA test.

Race. Prostate cancer is more common in some racial and ethnic groups than in others and medical experts do not know why. About 4 out of 100 African American men will die from prostate cancer while about 3 out of 100 white men will die.

Family history. Men with a father or brother who had prostate cancer have a greater chance of getting it themselves. The risk for these men is twice as high as men who do not have a relative who has had prostate cancer.

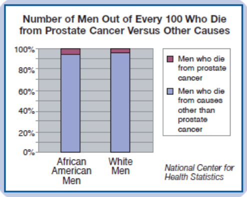

National Center for Health Statistics

Men Should Decide Whether They Feel the PSA Test is Right for Them and Talk With Their Doctors

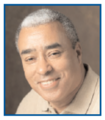

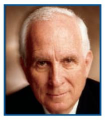

Many men think that PSA is just a simple blood test. But once you have the test, it can lead you quickly on a path to major treatment that you may or may not want.

If the biopsy shows you have prostate cancer, although it is most likely the harmless kind, there is no way to tell, so you will likely be encouraged by doctors to receive treatment.

Men say that it's hard to say "no" to the doctors' advice once they are diagnosed—even if the men don't want treatment.

> Think about whether you would want treatment before deciding whether the PSA test is right for you. If you would not want treatment, you should not get the PSA test in the first place.

Making Good Decisions Depends on Getting All the Facts

There are different decisions to be made about these three men's health issues. For:

- Heart attack and stroke—decide how you want to lower your risk factors.
- Colon cancer—decide which test to have.
- Prostate cancer—decide whether or not to get a PSA test.

Talk to your doctor about these decisions—they're important ones for you to make.

Men's Health Choices

	How much of a difference could this choice make in keeping me healthy?	How sure are doctors that it will help?
Reducing risk factors for heart attack and stroke	A lot	Very sure
Getting tested for colon cancer	Some	Very sure
Getting a PSA text	Some	Not sure

For more information go to www.menshealthdecisions.org.

You Decide

You Decide

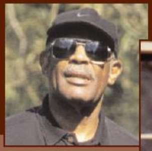

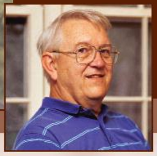

Men's Health Choices

Good health has a lot to do with making good decisions.

This brochure is to help you make decisions about three important health issues:

- Heart attack and stroke
- Colon cancer
- Prostate cancer

This brochure is for men ages 40-80.

You Decide

Heart Attack and Stroke

More than 50 out of 100 men die of heart attack and stroke. Your chances of having a heart attack and stroke are higher if you have certain “risk factors.” Some risk factors—like age, sex, or family history of heart attack and stroke—can’t be changed. But other risk factors can be changed.

The following risk factors are ones you can change to lower your chance of heart attack and stroke:

1. High blood pressure. Get your blood pressure checked at least yearly and get it treated if it’s high. There are many good treatments today that control blood pressure without giving you side effects.
2. High cholesterol. Get your cholesterol checked at least every 5 years and treat it if it’s high. Today’s treatments are very safe and work well.
3. Smoking. If you smoke, talk to your doctor about how to quit. Doctors have better ways than ever to help people stop smoking.
4. Poor diet and lack of physical activity. Eat healthy foods like fruits, vegetables, whole grains, and fish. If you are overweight, you should cut back on the number of calories you eat. Also get 20-30 minutes of physical activity at least 4 days a week.

For some people, taking a baby aspirin every day can lower your chance of having a heart attack. You should talk to your doctor about whether this is a good idea for you.

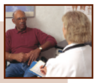

Doctors are very sure that men who don’t smoke and who keep their blood pressure and cholesterol levels under control will lower their chances of heart attack and stroke ***a lot***. Talk to your doctor and others about how you can do this, and how you can reduce other risk factors for heart attack and stroke.

Colon Cancer

Although it is not as common as heart attack and stroke, colon cancer is an important problem for middle-aged and older men. About 2 in every 100 men will die from colon cancer. Doctors don’t know yet what to tell people to do or not to do to keep them from getting colon cancer.

Doctors do know that tests to find colon cancer early can lower your chance of dying from it. These tests find both early cancer and polyps. Polyps are small tags of skin on your colon that can sometimes turn into cancer.

Tests for colon cancer

There are several tests that can find polyps and even colon cancer early, when treatment is most effective. It’s a good idea to get one of these tests on a regular basis beginning at age 50. Some tests are better at finding cancer, but more difficult for the patient. Other tests are easier, but need to be done more often.

1. Cards for blood in the stool. These are cards you take home and smear with your bowel movements, then send back to the doctor to check for hidden blood. If there is blood in your stool, you may need more tests. This test should be done every year.
2. Sigmoidoscopy (sig-moyd-ah-sco-pee). In this test, a doctor puts a small, flexible tube with a light in your rectum to look for small cancers or polyps. It goes about half way into your colon. This test should be done every 5 years.
3. Colonoscopy (co-lawn-ah-sco-pee). This test is like the sigmoidoscopy except that the tube goes all the way into your colon. It can find more cancers, but you will need more time to prepare for the test and recover from it. The test also takes longer. This test should be done every 10 years.

Doctors are very sure that having a test to find early colon cancer will help some men to live longer. Men should decide what test to have to find early colon cancer. Talk to your doctor about what test is best for you.

Prostate Cancer

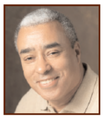

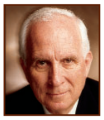

Like colon cancer, prostate cancer is not as common a problem as heart attack and stroke. The number of men dying of prostate cancer each year is nearly the same as the number dying of colon cancer. About 3 out of every 100 white men and 4 out of every 100 African-American men will die of prostate cancer. Like colon cancer, doctors don’t know yet what to tell people to do or not to do to keep them from getting prostate cancer.

The Prostate Specific Antigen (or PSA) test is a blood test that can help find prostate cancer. A small amount of PSA in the blood is normal. Higher amounts of PSA can come from prostate cancer or from having an enlarged prostate with no cancer (a condition that many men have after age 50). That is why most men with a high PSA level don’t have cancer – they have an enlarged prostate instead. Also, some prostate cancers don’t make much PSA – so some men have prostate cancer even though they have a normal PSA. So, the PSA test has some problems.

Many men think that the PSA is just a simple blood test. But once you have the test, it can lead you quickly on a path to major treatment that you may or may not want.

There Are Two Types of Prostate Cancer – Harmless and Dangerous

The prostate changes as men get older. In some men, the prostate gets larger. Another change is that some normal parts of the prostate start to look like cancer. Even though they look like cancer, these parts don't act like cancer – they never cause any problems. These are called "harmless" prostate cancers. Men can live long, normal lives with a harmless prostate cancer. Research shows that harmless prostate cancers grow so slowly that they will never become dangerous.

Most prostate cancers are harmless. If you think of 10 men who have prostate cancer, about 6 have harmless cancer. Only about 4 of these 10 men have the dangerous type of prostate cancer.

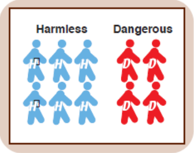

How can we find prostate cancer early? What are its symptoms?

Early prostate cancer doesn't have any symptoms – so that's not a good way to find it. The best way would be if doctors could just find dangerous prostate cancers and leave the harmless ones alone. But the PSA test finds both kinds of prostate cancers – harmless and dangerous – and doctors can't tell which one a man has.

6

Different Treatments for Prostate Cancer

If your PSA test level is high, your doctor will probably send you to a specialist. The specialist will do a "biopsy" of your prostate (a biopsy is when they stick small needles in your prostate to take samples to do more tests.)

If the biopsy shows cancer, it means that you would probably get treatment. Common treatments are:

- surgery (radical prostatectomy)—a major operation that removes the entire prostate, and
- external radiation therapy—burning a part of the prostate by using radiation.

Doctors don't know which treatment for early prostate cancer is best or if any of today's treatments help men live longer. Doctors do know that about half of all men who get surgery or radiation treatment will have permanent side effects from that treatment, including problems having an erection (also called "impotence") and problems holding their urine (also called "incontinence").

A problem with the PSA test is that it leads some men with harmless prostate cancer to get treatment that they don't need.

7

B. Values Clarification Exercise

A second purpose of our coaching tool was to help men clarify their values. We helped men clarify their values for prostate cancer screening through a process of rating and ranking several decision factors.

Our values clarification process is outlined below:

1) Men read a series of two opposing statements about five decision factors (see table 1 below)
2) Men chose which of the two statements best represented their own feelings
2) Men ranked the statements in order of influence to their decision making
3) Based on the color-coding of cards (red=against screening; blue=for screening), men were encouraged to consider whether or not they wanted to be screened.

Table 1. Factors and Feelings Influencing the Decision about Prostate Cancer Screening

Decision Factor	Statement Favoring Screening (in red)	Statement Against Screening (in blue)
**The chances of dying from prostate cancer**	I have a high chance of dying from prostate cancer, even compared with dying from other causes.	I have very little chance of dying from prostate cancer compared with my chance of dying from other causes.

**The need to know**	I don't like the idea of having prostate cancer and not knowing it even though more prostate cancers are harmless than are dangerous.	I'm okay with *not* knowing I have prostate cancer because more prostate cancers are harmless than dangerous.
**The certainty of the PSA test**	The fact that the PSA test doesn't give me a definite answer about whether I do or don't have cancer does not bother me; nothing in life is 100%.	I would only want to have the PSA test if it could tell me for sure if I do or don't have cancer. Because the PSA test can't do that, I don't like the idea of having it.
**The certainty of treatment benefit**	I don't mind having a serious treatment (like major surgery or radiation for prostate cancer) even if doctors can't tell me if it can help or not.	I don't like the idea of having a serious treatment (like major surgery or radiation for prostate cancer) if doctors can't tell me if it can help me or not.
**Worry over treatment side effects**	Getting the side effects from treating the prostate cancer (permanent trouble having sex or holding my urine) doesn't sound so bad to me.	Getting the side effects from treating prostate cancer (permanent trouble having sex or holding my urine) would really bother me a lot.

C. Preparing for discussions with the doctor

A final purpose of our coaching tool was to help men prepare for discussions with their doctor.

Our preparation process is outlined below:

1) Men identified barriers they have in discussing things with their doctor from among a list of common barriers (see table 2)
2) Scripted counseling was provided in response to each barrier identified (see table 2)
3) Each men is provided with a "list pad" (see below), which summarizes key messages and encourages them to write down questions to ask their doctor

Table 2. Common Barriers to Participation in Decision Making and Ways to Overcome Them

Barriers to Participation in Decision Making	Scripted Advice to Overcome Barrier
**I am not comfortable asking my doctor questions about my medical care**	Many people feel that way but it is okay to ask your doctor questions. Most doctors expect their patients to ask questions when they want

	to know something. Your doctor likely sees it as an important part of his/her job to inform his/her patients about medical issues. Can you think of a question right now that you'd like to ask your doctor? Planning ahead can make asking your doctor questions easier. You might also use this pad. [example list pad provided below]
**I could upset my doctor by giving my opinions about having a PSA test.**	Most doctors see it as part of their job to listen to what you think about your medical care. In fact, for a lot of health care today, the doctor needs to know what you think to give you the best care s/he can. Your doctor has to rely on you to let him/her know what you do and don't want, and when things are or are not working for you. You are the only one who can give him/her that important medical information. Are you thinking right now of an opinion you have about the PSA test that might be hard to say to your doctor? If so, why don't we talk about it and plan how you'll say it to your doctor. Planning ahead can make saying your opinions about the PSA test easier.
**It would be hard for me to interrupt my doctor to bring up the PSA test.**	If you are not comfortable interrupting the doctor or find it hard to break in, one thing that can help is to get your concerns on the agenda for the visit at the beginning. At the end of our meeting, I will give you a copy of a form that sums up what you have told me. Many men find it helpful to give this form to the doctor to help start a conversation about some of their concerns. It helps to do this upfront, at the very beginning of the visit, so your doctor can plan the time that you have together accordingly
**I don't feel it's my place to disagree with my doctor about the PSA test**	Most doctors see it as part of their job to listen to what you think about your medical care, even if you disagree with him/her. In fact, for a lot of health care today, the doctor needs to know what you think to be able to give you the best care s/he can. Your doctor relies on you to let him/her know what you do and don't want, and when things are or are not working for you. Sometimes that will mean disagreeing with him/her. That's expected to happen sometimes. You are the only one who can give

	him/her that important medical information.
**I worry that I will take up too much of my doctor's time if I raise my own concerns or questions about the PSA test.**	Most doctors see it as part of their job to listen to what you think about your medical care and to answer your questions. The visit is really for you so it is important that the time be used to focus on things that are important to you. Doctors are busy but they know that it is only by addressing your concerns that the time with you will be used most wisely. Some things that can help save time for your doctor when you have questions or concerns are:<br><br>1) Letting your doctor know upfront, right away, about your questions and concerns. That way she/he can plan the time you have together to take those needs into account. It can be harder for your doctor to make time for your concerns if he/she thinks that the visit is done and then have a lot of questions come up while they are on their way out the door. To get the most out of your visit, let your doctor know as soon as possible if there are things you want to talk about in a visit.<br><br>2) Another thing that can help you raise your questions and concerns is giving your doctor a form that sums up your feelings. It should help start a conversation about some of the things you want to talk over with your doctor.
**My doctor uses medical words that I don't understand when we talk about things like the PSA test.**	Most doctors try to speak in plain English but many doctors sometimes forget and slip into medical talk without realizing it because it is so familiar to them. Your doctor will likely appreciate your letting him/her know if s/he is doing this. It's okay to just ask your doctor to slow down a little and speak in plain English. It won't be the first time. Can you think of what you might say to your doctor when your doctor uses words you don't understand? Planning ahead about what you'll say can help you get the information you'll need from your doctor.
**I don't like to ask my doctor to explain things better because I'm embarrassed to admit that I did not understand what s/he said.**	You should not be embarrassed if you don't understand what your doctor says all the time. Everyone has their own style of communicating and they don't always match

	each other. Most doctors take the time to explain things to you because they want you to understand things about your health. Many medical issues are complicated and take time to understand. Your doctor understands this and expects people to ask questions to help clear up what s/he is saying. When you do this, you are helping your doctor know how to best get the message across to you. Can you think of what you might say to your doctor to let him know he needs to be clearer about what he wants you to do? Planning ahead about what you'll say can help you get the information you'll need from your doctor.

Sample List Pad (Prostate Only Version)

Men's Health Choices

Deciding About the PSA Test

1. **First, get the facts.** Check out the brochure and website, www.u-decide.org, that go along with this list.
2. **Next, think about what you want.** Is the PSA test right for you or not? Remember Joe and Frank? See the two-sided card.
3. **Then, talk with your doctor. Tips:**
   - Before your visit, make a list of any questions or concerns you have about your health. Use the other side of this pad to write them down.
   - Show your list to your doctor when he or she first comes into the room.
   - Tell your doctor up front that you want to be sure to talk about the PSA test as well as any other questions on your list.
   - If you don't understand, it's okay to ask your doctor to slow down or to put things in plain English.

For more information:
www.u-decide.org

You Decide

Things to talk with my doctor about:

My main reason for coming to the doctor today:

____

____

Other things on my mind are:

1. ______________________________
2. ______________________________
3. ______________________________
4. ______________________________
5. **Whether or not to get a PSA test**
   Doctor, I want to tell you how prostate cancer looks from where I stand. I am more like ____________ (Frank or Joe).

   I want to protect myself against prostate cancer, but don't want to have problems with having sex or holding my urine (water). How do I decide? What should I think about?

____

____

You Decide
