# Supplementary material for: Shared decision making for prostate cancer screening: the results of a combined analysis of two practice-based randomized controlled trials
Source: BMC Med Inform Decis Mak. 2012 Nov 13;12:130. doi: 10.1186/1472-6947-12-130 (PMC3582602; doi:10.1186/1472-6947-12-130)
Supplement: Additional file 3 — The Effect of the Intervention on Changes in Key Components of Decision Making. [file 1472-6947-12-130-S3.docx]

# **Additional file 3**

|  | **Control,**  **% (n)**  (n=70) | **Intervention,**  **% (n)**  (n=58) | **Unadjusted Absolute Difference**  **(95% CI)*** | **RR**  **(95% CI)†** | | **RR**  **(95% CI)** ‡ |
| --- | --- | --- | --- | --- | --- | --- |
| Overall | | | | | | |
| **PSA is a Decision:**  **Subgroups of Men (dis)agreeing across study** |  |  |  |  |  | |
| % Men who disagreed throughout study | 73% (51) | 31% (18) | -- | -- |  | |
| % Men who disagreed after intervention only | 4% (3) | 5% (3) | -- | -- |  | |
| % Men who agreed throughout study | 13% (9) | 29% (17) | -- | -- |  | |
| *% Men who agreed after intervention only* | 10% (7) | 34% (20) | 24%  (10 to 38%) | 5.56  (2.85 to 100) | 3.44  (1.70 to 5.76) | |
| **Key Knowledge:**  **Subgroups of Men (not) having key knowledge across study:** |  |  |  |  |  | |
| Never had key knowledge | 84% (58) | 53% (30) | -- | -- |  | |
| Key knowledge at baseline only | 3% (2) | 0% (0) | -- | -- |  | |
| Key knowledge throughout study | 7% (5) | 4% (2) | -- | -- |  | |
| *Key knowledge after intervention only* | 6% (4) | 44% (25) | 38%  (24% to 52%) | 9.09  (4.17 to ∞) | 8.06  (3.56 to 13.37) | |
| **Among Men Who Talked with Their Doctor About PSA Testing** | | | | | | |
|  | **Control,**  **% (n)**  (n=51) | **Intervention,**  **% (n)**  (n=38) | **Absolute Difference**  **(95% CI)*** | **Adjusted RR**  **(95% CI)†** | | **Adjusted RR**  **(95% CI) §** |
| **% Participation at preferred level, by post intervention desire for participation**†**:** |  |  |  |  | |  |
| Preferred to Make Decision after the intervention: | 55% (6/11) | 50% (7/14) | -5%  (-43% to 35%) | 0.96  (0.58 to 2.7) | | 1.58  (0.91 to 2.41) |
| Preferred Shared Decision after the intervention: | 85% (33/39) | 87% (20/23) | 2%  (-15% to 20%) | 1.04  (0.82 to 1.45) | | 0.81  (0.56 to 1.03) |
| Preferred MD to Make Decision after the intervention: | 0% (0/1) | 0% (0/1) | 0% | --- | |  |

**Table 1. The Effect of the Intervention on Changes in Key Components of**

**Decision Making**

*Pearson Chi-square tests

† Adjusted for random effects of physician

‡ Adjusted for random effects of physician and practice
